# Rationale, design, and baseline characteristics of the CArdiovascular safety and Renal Microvascular outcomE study with LINAgliptin (CARMELINA^®^): a randomized, double-blind, placebo-controlled clinical trial in patients with type 2 diabetes and high cardio-renal risk

**DOI:** 10.1186/s12933-018-0682-3

**Published:** 2018-03-14

**Authors:** Julio Rosenstock, Vlado Perkovic, John H. Alexander, Mark E. Cooper, Nikolaus Marx, Michael J. Pencina, Robert D. Toto, Christoph Wanner, Bernard Zinman, David Baanstra, Egon Pfarr, Michaela Mattheus, Uli C. Broedl, Hans-Juergen Woerle, Jyothis T. George, Maximilian von Eynatten, Darren K. McGuire, CARMELINA® investigators

**Affiliations:** 1 Dallas Diabetes Research Center at Medical City, 7777 Forest Lane, Suite C-685, Dallas, TX 75230 USA; 2 The George Institute for Global Health, Faculty of Medicine, University of New South Wales, Sydney, NSW Australia; 3 Duke Clinical Research Institute, Duke Health, Durham, NC USA; 4 Head of Diabetes, Monash University, Melbourne, VIC Australia; 5 Department of Internal Medicine I, University Hospital Aachen, RWTH Aachen University, Aachen, Germany; 6 University of Texas Southwestern Medical Center, Dallas, TX USA; 7 Dept of Medicine, Würzburg Univ Clinic, Würzburg, Germany; 8 Lunenfeld-Tanenbaum Research Institute, Mount Sinai Hospital, Toronto, Canada; 9 University of Toronto, Toronto, Canada; 10 Boehringer Ingelheim bv, Alkmaar, The Netherlands; 11 Boehringer Ingelheim Pharma GmbH & Co. KG, Ingelheim, Germany; 12 Ulm University, Ulm, Germany; 13 Division of Cardiology, Department of Internal Medicine, University of Texas Southwestern Medical Center, Dallas, TX USA

**Keywords:** Diabetes mellitus, type 2, Cardiovascular diseases, Diabetic nephropathies, Dipeptidyl-peptidase IV inhibitors, Linagliptin, Clinical trial, phase IV, Research design, Treatment outcome

## Abstract

**Background:**

Cardiovascular (CV) outcome trials in type 2 diabetes (T2D) have underrepresented patients with chronic kidney disease (CKD), leading to uncertainty regarding their kidney efficacy and safety. The CARMELINA^®^ trial aims to evaluate the effects of linagliptin, a DPP-4 inhibitor, on both CV and kidney outcomes in a study population enriched for cardio-renal risk.

**Methods:**

CARMELINA^®^ is a randomized, double-blind, placebo-controlled clinical trial conducted in 27 countries in T2D patients at high risk of CV and/or kidney events. Participants with evidence of CKD with or without CV disease and HbA1c 6.5–10.0% (48–86 mmol/mol) were randomized 1:1 to receive linagliptin once daily or matching placebo, added to standard of care adjusted according to local guidelines. The primary outcome is time to first occurrence of CV death, non-fatal myocardial infarction, or non-fatal stroke. The key secondary outcome is a composite of time to first sustained occurrence of end-stage kidney disease, ≥ 40% decrease in estimated glomerular filtration rate (eGFR) from baseline, or renal death. CV and kidney events are prospectively adjudicated by independent, blinded clinical event committees. CARMELINA^®^ was designed to continue until at least 611 participants had confirmed primary outcome events. Assuming a hazard ratio of 1.0, this provides 90% power to demonstrate non-inferiority of linagliptin versus placebo within the pre-specified non-inferiority margin of 1.3 at a one-sided α-level of 2.5%. If non-inferiority of linagliptin for the primary outcome is demonstrated, then its superiority for both the primary outcome and the key secondary outcome will be investigated with a sequentially rejective multiple test procedure.

**Results:**

Between July 2013 and August 2016, 6980 patients were randomized and took ≥ 1 dose of study drug (40.6, 33.1, 16.9, and 9.4% from Europe, South America, North America, and Asia, respectively). At baseline, mean ± SD age was 65.8 ± 9.1 years, HbA1c 7.9 ± 1.0%, BMI 31.3 ± 5.3 kg/m^2^, and eGFR 55 ± 25 mL/min/1.73 m^2^. A total of 5148 patients (73.8%) had prevalent kidney disease (defined as eGFR < 60 mL/min/1.73 m^2^ or macroalbuminuria [albumin-to-creatinine ratio > 300 mg/g]) and 3990 patients (57.2%) had established CV disease with increased albuminuria; these characteristics were not mutually exclusive. Microalbuminuria (n = 2896 [41.5%]) and macroalbuminuria (n = 2691 [38.6%]) were common.

**Conclusions:**

CARMELINA^®^ will add important information regarding the CV and kidney disease clinical profile of linagliptin by including an understudied, vulnerable cohort of patients with T2D at highest cardio-renal risk.

*Trial registration* ClinicalTrials.gov identifier—NCT01897532; registered July 9, 2013

**Electronic supplementary material:**

The online version of this article (10.1186/s12933-018-0682-3) contains supplementary material, which is available to authorized users.

## Background

People with type 2 diabetes (T2D) are at increased risk for both cardiovascular (CV) disease and microvascular complications such as chronic kidney disease (CKD) and kidney failure. In 2008, concerns about adverse CV events associated with the peroxisome proliferator-activated receptor agonists rosiglitazone [1] and muraglitazar [2] were among the issues that led the US Food and Drug Administration (FDA) and European Medicines Agency (EMA) to mandate that novel glucose-lowering drugs for treatment of T2D demonstrate CV safety [3–5]. The CV outcome trials conducted in response to this guidance over the past decade have consequently focused on T2D patients at high risk for CV complications [6–16]. In contrast, evaluation of novel glucose-lowering drugs in individuals at high risk of adverse kidney outcomes has been sparse and relatively neglected.

**Fig. 1:**
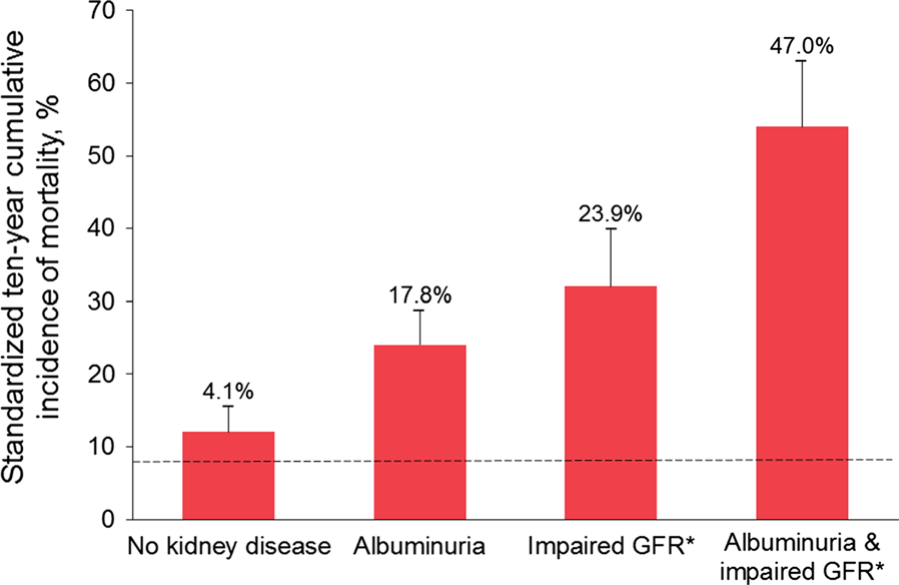
10-year mortality in T2D by kidney disease manifestation in the United States. The dashed line indicates mortality in persons without diabetes or kidney disease (the reference group, 7.7%). The numbers above bars indicate excess mortality above the reference group. Error bars indicate upper limits of the 95% confidence intervals. Republished with permission of the American Society of Nephrology from Afkarian et al. [21]; permission conveyed through Copyright Clearance Center, Inc. *Impaired GFR was defined as GFR ≤ 60 mL/min/1.73 m^2^. *GFR* glomerular filtration rate, *T2D* type 2 diabetes

Approximately 50% of patients with T2D globally also have some evidence of CKD [17], which is associated with significantly increased risk of progression to end-stage kidney disease (ESKD) and premature mortality (Fig. 1). CKD is also one of the strongest risk factors for CV events [18]. A 2016 summit convened by the International Society of Nephrology concluded that a concerted effort is required to increase the quantity and quality of clinical trials investigating CKD [19]; however, there are notable challenges involved in conducting such studies [20]. The paucity of clinical trials specifically designed to evaluate kidney-related efficacy and safety outcomes with glucose-lowering drugs represents an important gap in knowledge to support informed treatment decision-making in patients with T2D at high risk for kidney complications.

Dipeptidyl peptidase-4 (DPP-4) inhibitors are now established as oral glucose-lowering drugs with little intrinsic risk of causing hypoglycemia or weight gain [22]. The DPP-4 inhibitors evaluated to date in CV outcomes studies (saxagliptin, alogliptin, sitagliptin) have demonstrated CV safety with regard to atherosclerotic CV disease outcomes, with neutral effects on major adverse CV events compared with placebo [6–8]. However, the incidence of hospitalization for heart failure was statistically increased in the SAVOR-TIMI 53 trial of saxagliptin versus placebo [6] and numerically increased in the EXAMINE trial of alogliptin versus placebo [23]; whereas no effect on the incidence of heart failure hospitalization was observed in the TECOS trial of sitagliptin versus placebo [24]. These observations have prompted FDA product label warnings in the US for all members of the DPP-4 inhibitor class.

Linagliptin is a DPP-4 inhibitor that is excreted predominantly by non-renal pathways, unlike most other members of this drug class, and thus can be prescribed to patients with T2D at a single dose irrespective of kidney function [25, 26]. In pooled analyses of clinical trials designed to evaluate glycemic efficacy and tolerability over the short term (typically 12–24 weeks), linagliptin was not associated with an increase in either CV [27] or kidney risk [28] in patients with T2D, but was associated with a significant reduction in clinically relevant kidney disease endpoints [28].

The CV and kidney clinical profile of linagliptin is being comprehensively evaluated in the CArdiovascular safety and Renal Microvascular outcomE study with LINAgliptin (CARMELINA^®^) trial. Uniquely among the outcomes studies of glucose-lowering drugs initiated to date, the CARMELINA^®^ trial was designed to recruit patients with T2D at high risk of both CV and kidney complications who had evidence of compromised kidney function with or without CV disease. We report here the study design and methods of CARMELINA^®^ alongside the pooled baseline clinical characteristics of the patients in this trial.

## Methods

### Study design

**Fig. 2:**
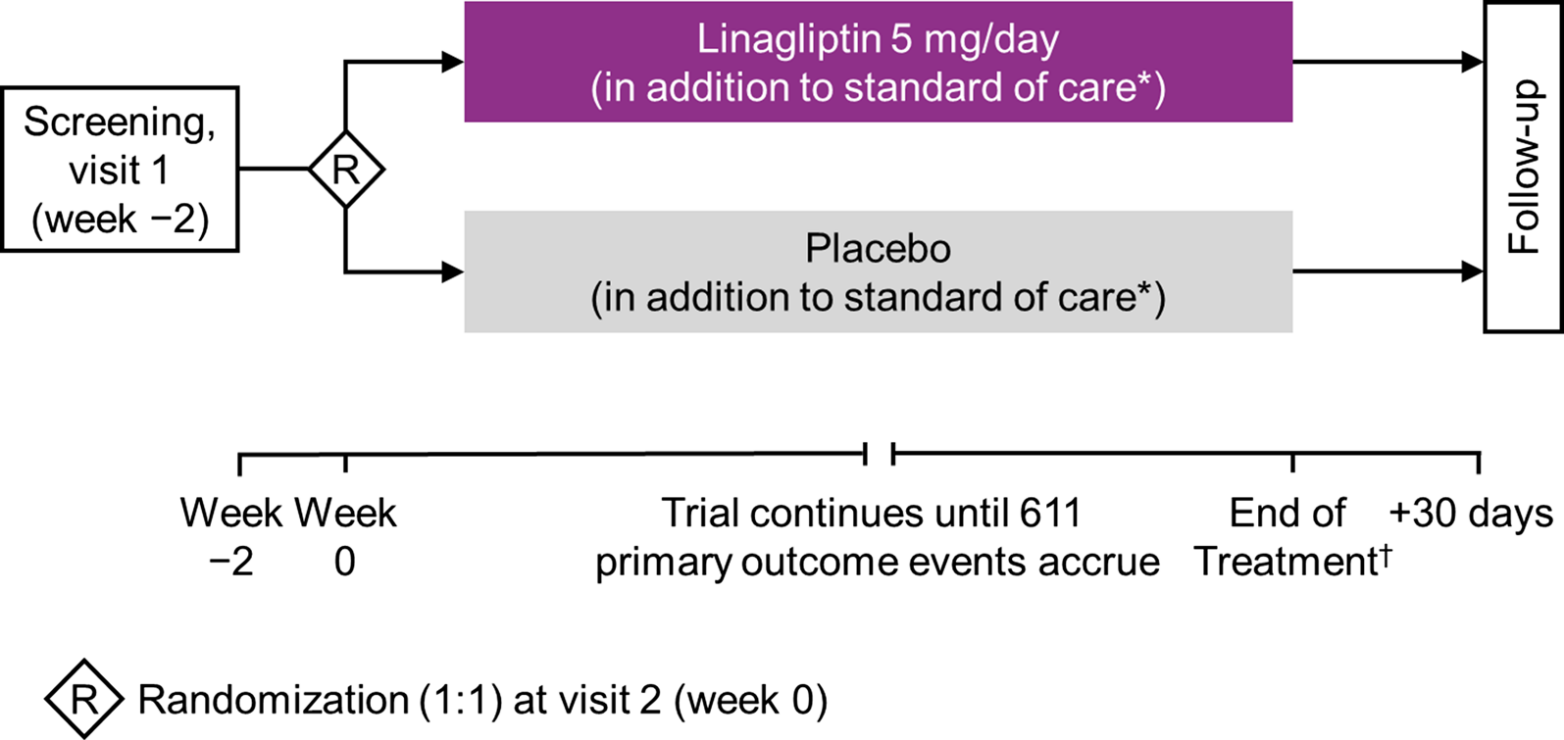
Design of the CARMELINA^®^ trial. *Additional glucose-lowering therapy may be given on top of study medication if HbA1c > 7.5%; investigators are encouraged to treat all other CV risk factors in accordance with local or regional standards of care. ^†^Participants who stop study drug early are observed until study end (not just until 30 days after the end of treatment with study drug). *CV* cardiovascular, *HbA1c* glycated hemoglobin A1c

The CARMELINA^®^ study is a multi-national, randomized, double-blind, placebo-controlled clinical trial conducted in 27 countries (ClinicalTrials.gov identifier: NCT01897532) (Fig. 2). CARMELINA^®^ is an event-driven trial designed to assess the impact of linagliptin versus placebo on CV and kidney outcomes in a population of patients with T2D enriched for both macrovascular and kidney microvascular risk. The study is designed to run until at least 611 participants have had an adjudicated primary-outcome event. The study protocol was approved by institutional review boards, independent ethics committees and competent authorities according to national and international regulations. CARMELINA^®^ was conducted in accordance with the ICH Harmonised Tripartite Guideline for Good Clinical Practice. All participants provided written informed consent prior to entering the study.

### Participants

Patients with T2D aged ≥ 18 years with glycated hemoglobin A1c (HbA1c) 6.5–10.0% (48–86 mmol/mol) and body-mass index (BMI) ≤ 45 kg/m^2^ were eligible for inclusion. Participants had to be at high risk of vascular events based on established history of CV disease, and/or the presence of markers of CKD (Table 1). Participants could be either drug-naïve or receiving any glucose-lowering therapy except glucagon-like peptide (GLP)-1 receptor agonists, DPP-4 inhibitors and/or sodium-glucose co-transporter 2 (SGLT2) inhibitors. Those individuals already receiving glucose-lowering therapy had to be on the same dose for at least 8 weeks prior to randomization.

**Table 1:** CARMELINA^®^ inclusion criteria

High risk of vascular events (I and/or II)		
I.	Albuminuria (UACR ≥ 30 mg/g or ≥ 30 μg albumin/min or ≥ 30 mg albumin/24 h in two out of three unrelated spot urine or timed samples in the 24 months prior to randomization) and previous macrovascular disease, defined as one or more of the following	
	(a)	Confirmed history of myocardial infarction (> 2 months prior to screening)
	(b)	Advanced coronary artery disease, defined by any one of the following
		≥ 50% narrowing of the luminal diameter in 2 or more major coronary arteries (left anterior descending, circumflex, right coronary artery) by coronary angiography, MRI angiography or CT angiography
		Left main stem coronary artery with ≥ 50% narrowing of the luminal diameter by coronary angiography, MRI angiography or CT angiography
		Prior percutaneous or surgical revascularization of ≥ 2 major coronary arteries ≥ 2 months prior to screening
		The combination of prior percutaneous or surgical revascularization of 1 major coronary artery ≥ 2 months prior to screening, and ≥ 50% narrowing of the luminal diameter by coronary angiography, MRI angiography or CT angiography of at least 1 additional major coronary artery
	(c)	High-risk single-vessel coronary artery disease, defined as the presence of ≥ 50% narrowing of the luminal diameter of one major coronary artery by coronary angiography, MRI angiography or CT angiography in patients not revascularized and at least one of the following
		A positive non-invasive stress test, confirmed by either
		A positive ECG exercise tolerance test in patients without left bundle branch block, Wolff–Parkinson–White syndrome, left ventricular hypertrophy with repolarization abnormality, or paced ventricular rhythm, atrial fibrillation in case of abnormal ST-T segments
		A positive stress echocardiogram showing induced regional systolic wall motion abnormalities
		A positive nuclear myocardial perfusion imaging stress test showing stress-induced reversible perfusion abnormality
		A positive cardiac stress perfusion MRI showing a stress-induced perfusion defect
		Patient discharged from hospital with a documented diagnosis of unstable angina pectoris between 2 and 12 months prior to screening
	(d)	History of ischemic or hemorrhagic stroke (> 3 months prior to screening)
	(e)	Presence of carotid artery disease (symptomatic or not) documented by either
		Imaging techniques with at least one lesion estimated to be ≥ 50% narrowing of the luminal diameter
		Prior percutaneous or surgical carotid revascularization
	(f)	Presence of peripheral artery disease documented by either
		Previous limb angioplasty, stenting or bypass surgery
		Previous limb or foot amputation due to macrocirculatory insufficiency
		Angiographic evidence of peripheral artery stenosis ≥ 50% narrowing of the luminal diameter in at least one limb (definition of peripheral artery: common iliac artery, internal iliac artery, external iliac artery, femoral artery, popliteal artery)
II	Impaired renal function with/without CV comorbidities	
		eGFR (MDRD) of 15 to < 45 mL/min/1.73 m^2^ at screening
		eGFR (MDRD) ≥ 45 to 75 mL/min/1.73 m^2^ at screening with UACR > 200 mg/g or > 200 μg albumin/min or > 200 mg albumin/24 h in two out of three unrelated spot urine or timed samples in the 24 months prior to randomization

*CT* computed tomography, *CV* cardiovascular, *ECG* electrocardiogram, *eGFR* estimated glomerular filtration rate, *MDRD* Modification of Diet in Renal Disease study equation, *MRI* magnetic resonance imaging, *UACR*, urinary albumin-to-creatinine ratio

Individuals who had had an acute coronary syndrome in the 2 months prior to screening were ineligible, as were those who had had a stroke or transient ischemic attack in the 3 months before screening, and those who were scheduled to have percutaneous coronary intervention (PCI) or coronary artery bypass graft surgery (CABG) or had had PCI and/or CABG in the 2 months before screening. Also excluded were individuals with ESKD, defined as an estimated glomerular filtration rate (eGFR) by the Modification of Diet in Renal Disease study equation (MDRD) of < 15 mL/min/1.73 m^2^ and/or the receipt of maintenance dialysis. Premenopausal women who were pregnant, nursing, or not practicing birth control were also excluded, as were individuals with active liver disease or impaired hepatic function (serum levels of alanine aminotransferase, aspartate aminotransferase or alkaline phosphatase equal to or greater than three times the upper limit of normal [≥ 3× ULN]), and those with prior or planned bariatric surgery. The full inclusion and exclusion criteria are shown in Additional file 1.

### Randomization, investigational product administration and follow-up

Eligible individuals were randomized 1:1 via an interactive telephone/web-based system to receive once-daily oral treatment with linagliptin 5 mg (the licensed dose) or matching placebo in a double-blind manner (Fig. 2). Treatment assignment was determined by computer-generated random sequence with stratification by geographical region. Following randomization, participants were instructed to return to the clinic for check-up after 12 weeks and then every 24 weeks until study end. Assessments included checks for adverse events and outcome events, physical examinations, vital signs, laboratory parameters, and 12-lead electrocardiograms (ECGs).

The use of additional medication for optimizing glycemic control according to local standard of care was encouraged throughout the trial independent of study treatment assignment, and included any approved glucose-lowering drugs except DPP-4 inhibitors, GLP-1 receptor agonists, and SGLT2 inhibitors. Dosing was required to adhere to local labelling and be in accordance with local/international guidelines. Intensification of glycemic control was advised for participants with fasting plasma glucose > 180 mg/dL (> 10.0 mmol/L) (confirmed by at least two measurements on different days) and those with HbA1c > 7.5% (> 58 mmol/mol). Investigators were also encouraged to treat all other CV risk factors (e.g. dyslipidemia, hypertension, albuminuria, smoking) in accordance with optimal local or regional guidelines and standards of care. Ultimately, changes in medication were at the discretion of the investigator and/or treating clinician.

### Outcomes and adjudication

The primary outcome is the time to the first occurrence of CV death, non-fatal myocardial infarction (MI) or non-fatal stroke—the so-called 3-point major adverse CV events (3P-MACE) composite outcome. The adoption of 3P-MACE as the primary outcome resulted from a study protocol amendment in 2016 from the original primary outcome of 4P-MACE (3P-MACE plus hospitalization for unstable angina). Although no longer a component of the primary composite, hospitalization for unstable angina continued to be an adjudicated endpoint. This amendment was made by the CARMELINA^®^ steering committee based on emerging evidence from other CV outcomes studies accumulated since study start indicating that 3P-MACE is the more suitable primary outcome for studies of glucose-lowering medications [29] and, accordingly, is now preferred by the FDA as a CV outcome over the 4P-MACE composite outcome [30]. This change aligns CARMELINA^®^ with other recent and ongoing studies of novel glucose-lowering drugs [29]. The steering committee had neither access to unblinded data nor input from the independent data monitoring committee when making this amendment, and the study remained fully blinded to the steering committee, sponsor, trial team, investigators and participants during these deliberations.

The key secondary outcome is a kidney composite of time to first occurrence of sustained ESKD, renal death (adjudicated death due to kidney disease), or a sustained decrease of ≥ 40% in eGFR from baseline. This kidney composite outcome had been amended in 2016 to lower the threshold for sustained eGFR decrease from ≥ 50 to ≥ 40%. This amendment was made on the basis of evolving clinical evidence and multi-disciplinary workshops convened by the US National Kidney Foundation and the FDA, where it was concluded that there is strong evidence for a sustained decline of ≥ 40% in eGFR being an appropriate outcome for clinical trials seeking to identify evidence of kidney protection [31, 32]. Furthermore, both the FDA and the EMA have acknowledged that a ≥ 40% decline in eGFR is a suitable outcome in kidney outcome studies to improve trial efficiency [32, 33]. The original key secondary composite outcome (time to first occurrence of ESKD, renal death, or a sustained decrease of ≥ 50% in eGFR from baseline) will be evaluated as a tertiary endpoint.

Further tertiary outcomes include the following, among others: 4P-MACE, CV death, fatal or non-fatal MI, fatal or non-fatal stroke, hospitalization for unstable angina, stent thrombosis, transient ischemic attack, all-cause mortality, and renal death. Additional kidney-related endpoints include transition in albuminuria class or CKD stage from baseline as well as eGFR slope analyses. Glucose-related outcomes include change from baseline in levels of HbA1c and fasting plasma glucose.

All CV and cerebrovascular events are prospectively ascertained and centrally adjudicated by an independent and blinded external clinical event committee, as recommended by the FDA [3]. Some heterogeneity in the risk of heart failure has been observed in previous outcome trials of DPP-4 inhibitors. In CARMELINA^®^, hospitalization for heart failure is prospectively evaluated. All reported events of hospitalization for heart failure are independently adjudicated under blinded conditions by a central committee based on pre-defined criteria. Hospitalization for heart failure is another pre-defined tertiary outcome in the protocol—both on its own and as a composite with CV death, consistent with contemporary best practice in heart failure trials. Separate independent and blinded clinical event committees adjudicate kidney and pancreatic events.

Identification of potential outcome events sent for adjudication based on investigator reports was supplemented using a search of the trial database for events based on Standardised Medical Dictionary for Regulatory Activities (MedDRA) Queries and other events defined in the adjudication charters to trigger adjudication committee review. Furthermore, all deaths were sent for adjudication. Additionally, a periodic, blinded pharmacovigilance review of all other (non-trigger) events was performed.

Safety is assessed based on all reported adverse events (including serious adverse events and adverse events of special interest), physical examinations, vital signs, laboratory parameters, and 12-lead ECGs. Hypoglycemia was defined as documented blood glucose ≤ 70 mg/dL (≤ 3.9 mmol/L) with the exception of severe hypoglycemia, which was defined as any episode requiring third-party assistance. Incidence and event rates will be analyzed for hypoglycemia ≤ 70 mg/dL (≤ 3.9 mmol/L) and < 54 mg/dL (< 3.0 mmol/L) or severe hypoglycemia. Adverse events pre-specified as being of special interest were hypersensitivity reactions, skin lesions, kidney adverse events including acute kidney injury, pancreatitis, pancreatic cancer, benign thyroid neoplasms, thyroid cancer and hepatic events. A periodic review of all lipase values ≥ 3× ULN not reported as adverse events was performed.

### Study oversight and organization

The CARMELINA^®^ trial was sponsored by Boehringer Ingelheim, the manufacturer of linagliptin, and Eli Lilly and Company. CARMELINA^®^ was designed jointly by independent academic investigators and sponsor-employed scientists and physicians with relevant clinical and methodological expertise, who together comprise the steering committee. The steering committee, led by the academic investigators, supervised the conduct of the trial, and an independent data monitoring committee regularly reviewed safety data, on the basis of which it recommended the trial to continue or terminate early according to a pre-specified charter. Independent contract research organizations were involved in interactive response technology for randomization, analyses of ECGs, blinded event adjudication, central laboratory analyses, and operational implementation of the trial.

### Statistical analysis

**Fig. 3:**
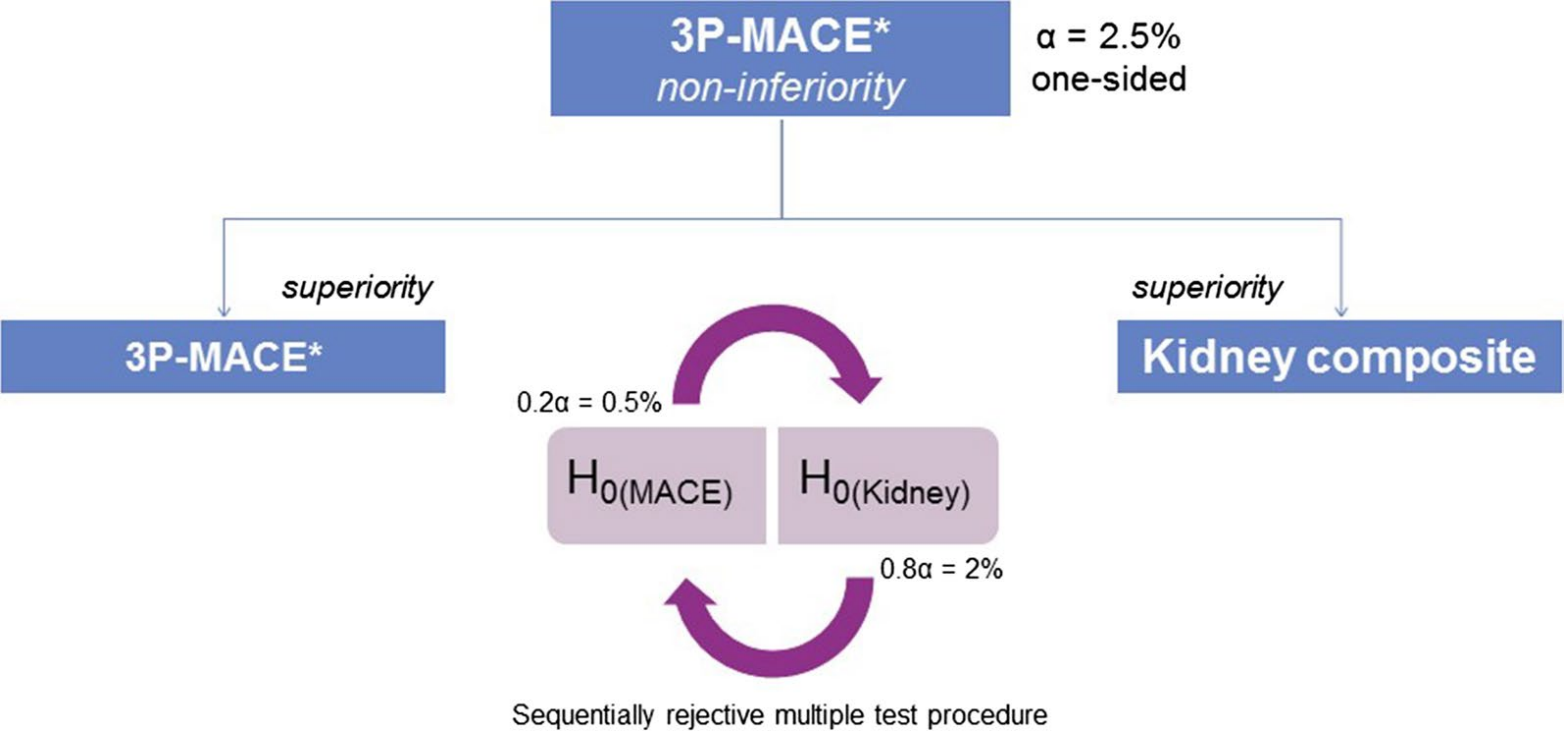
Statistical testing for the primary and secondary endpoints. For the final analysis, the first hypothesis (non-inferiority of the primary endpoint [3P-MACE]) will be tested at the one-sided alpha-level of 2.5%. In case of significance, the null hypothesis (H_0_) is rejected in a confirmatory sense and the next set of hypotheses (two separate hypothesis tests) will be tested: (a) test of the primary endpoint for superiority and (b) test of the composite renal endpoint for superiority. To adjust for multiplicity, a sequentially rejective multiple test procedure will be applied. Both one-sided hypotheses H_0(Sup1)_ and H_0(Sup2)_ will be tested separately, at the initial alpha-levels of ×0.2 alpha for the primary endpoint and ×0.8 alpha for the composite renal endpoint, respectively. If both null hypotheses cannot be rejected at these initial alpha-levels, the procedure stops and for neither endpoint can superiority be declared. After having shown superiority for one of these endpoints, the used alpha can be shuffled to the other test: If H_0(Sup2)_ is rejected at the alpha-level of ×0.8 alpha, then H_0(Sup1)_ can be tested at the full alpha-level of 2.5% (one-sided). If H_0(Sup1)_ is rejected at the alpha-level of ×0.2 alpha, then H_0(Sup2)_ can be tested at the full alpha-level of 2.5% (one-sided). *Cardiovascular death, non-fatal myocardial infarction, or non-fatal stroke. *H*_*0*_ null hypothesis, *3P*-*MACE* 3-point major adverse cardiovascular events

The primary null hypothesis was that treatment with linagliptin would be inferior by a hazard ratio of ≥ 1.3 compared with placebo as assessed by the time to first occurrence of any of the 3P-MACE (primary endpoint) events. Rejection of the null hypothesis will be evaluated by the upper limit of the two-sided 95% confidence interval (CI) for the hazard ratio, as required by FDA guidance [3]. The test of the primary outcome for non-inferiority is the first of the following two-step testing strategy in which superiority tests will only be performed if the first test is statistically significant: (i) non-inferiority test of the primary outcome; (ii) superiority tests of (a) the primary outcome and (b) the key secondary composite kidney outcome, with a sequentially rejective multiple test procedure (Fig. 3).

A total of 611 primary (3P-MACE) events provides 90% power to demonstrate non-inferiority of linagliptin versus placebo at the overall one-sided α-level of 2.5% if the hazard ratio is 1.0. The sample size and event estimates were based on the primary hypothesis.

If non-inferiority has been demonstrated, then superiority with regard to the primary outcome will be tested. Assuming 611 patients with an event and a hazard rate for linagliptin at any time 20% less than the risk for placebo, then the resulting power to demonstrate superiority at the final analysis is 79% for a test at the α-level of 2.5%.

Assuming 432 patients with a composite kidney outcome, an annualized event rate of 2.5% in the placebo group and a hazard rate for linagliptin of 0.75, then the resulting power to demonstrate superiority for the key secondary outcome at the final analysis is 85% if the test can be performed at the α-level of 2.5%.

The primary and key secondary outcomes will be analyzed using the treated set (all patients treated with at least one dose of study drug) with treatment assignment at randomization and including all adjudication-confirmed events that occur until study end. The primary and key secondary endpoints will be analyzed using a Cox proportional hazards regression model of time to the first event, with randomized treatment and geographical region as factors.

Sensitivity analyses will be conducted on the per-protocol set, which excludes patients with important protocol violations such as incorrect study drug taken; the on-treatment set with a minimum treatment duration of 30 days; and the treated set with censoring at 30 days after last dose of study drug (treated set + 30). For these analyses, events will be considered that have occurred no later than 30 days after last intake of study drug. An additional analysis will only include outcome events until last study drug intake (treated set + 0).

The primary and key secondary outcomes will also be analyzed in patient subgroups based on several baseline characteristics, including but not limited to the following: geographical region, age, sex, blood pressure, insulin use, eGFR < 60 mL/min/1.73 m^2^, and prevalent kidney disease (eGFR < 60 mL/min/1.73 m^2^ or urinary albumin-to-creatinine ratio [UACR] > 300 mg/g).

Safety will be evaluated in the treated set using descriptive statistical analyses of adverse events, as well as changes in clinical and laboratory parameters.

The data will be analyzed by the contract research organization in charge of study conduct, the sponsor, and the statistics group at the Duke Clinical Research Institute (Durham, NC, USA) who will conduct an independent statistical analysis.

## Results

Recruitment of participants for the CARMELINA^®^ study began in July 2013 and was completed in August 2016. A total of 12,280 individuals were screened and 6991 were randomized at 407 clinics in 27 countries. Of these, 6980 participants received at least one dose of study drug. Europe provided the largest number of participants (n = 2833 [40.6%]), followed by South America (n = 2310 [33.1%]), North America (n = 1180 [16.9%]) and Asia (n = 657 [9.4%]).

Pooled baseline characteristics of the participants are shown in Table 2. Participants had mean age, HbA1c, and BMI of 65.8 years, 7.9% and 31.3 kg/m^2^, respectively, with mean duration of diabetes of 14.7 years (Table 2). The majority were male (63%), white (80%) and had eGFR < 60 mL/min/1.73 m^2^ (62%). Mean eGFR was 54.6 mL/min/1.73 m^2^, and 1063 (15%) participants had severe renal impairment (eGFR < 30 and > 15 mL/min/1.73 m^2^). Median UACR was 162 mg/g (25th–75th percentile: 44–727 mg/g); 1390 participants (20%) were normoalbuminuric (UACR < 30 mg/g), while the majority had either microalbuminuria (UACR 30 − 300 mg/g; n = 2896 [41.5%]) or macroalbuminuria (UACR > 300 mg/g; n = 2691 [39%]). Mean systolic and diastolic blood pressure were 141 and 78 mmHg, respectively, and 95% of participants (6637) were taking antihypertensive medication. Angiotensin-converting enzyme (ACE) inhibitors or angiotensin-receptor blockers were being taken by 81% of the trial population, while 53% were taking diuretics.

**Table 2:** Baseline characteristics

	Total (n = 6980)
Age, years	65.8 ± 9.1
Male, n (%)	4390 (62.9)
Race, n (%)	
White	5595 (80.2)
Asian	641 (9.2)
Black/African American	411 (5.9)
Other^a^	333 (4.8)
Region, n (%)	
Europe	2833 (40.6)
South America	2310 (33.1)
North America	1180 (16.9)
Asia	657 (9.4)
Smoking status, n (%)	
Never smoker	3753 (53.8)
Ex-smoker	2507 (35.9)
Current smoker	713 (10.2)
Missing	7 (0.1)
eGFR (MDRD), mL/min/1.73 m^2^	54.6 ± 25.0
eGFR (MDRD), n (%)	
≥ 90 mL/min/1.73 m^2^	728 (10.4)
≥ 60 to < 90 mL/min/1.73 m^2^	1903 (27.3)
≥ 45 to < 60 mL/min/1.73 m^2^	1349 (19.3)
≥ 30 to < 45 mL/min/1.73 m^2^	1937 (27.8)
< 30 mL/min/1.73 m^2^	1063 (15.2)
UACR, mg/g, median (25th–75th percentile)	162 (44–727)^c^
UACR, n (%) [mg/g]	
< 30	1390 (19.9)
30–300	2896 (41.5)
> 300	2691 (38.6)
Missing	3 (0.0)
BMI, kg/m^2^	31.3 ± 5.3^d^
HbA1c, %	7.9 ± 1.0
Fasting plasma glucose, mmol/L^b^ (mg/dL)	8.4 ± 3.4 (151.8 ± 61.7)^e^
Diabetes duration, years	14.7 ± 9.5
Systolic blood pressure, mmHg	140.5 ± 17.9
Diastolic blood pressure, mmHg	77.8 ± 10.5
Total cholesterol, mmol/L (mg/dL)	4.5 ± 1.3 (172 ± 48)^f^
LDL cholesterol, mmol/L (mg/dL)	2.4 ± 1.0 (91 ± 40)^g^
HDL cholesterol, mmol/L (mg/dL)	1.2 ± 0.3 (45 ± 13)^h^
Triglycerides, mmol/L (mg/dL)	2.1 ± 1.5 (188 ± 133)^f^
Glucose-lowering therapy, n (%)	6802 (97.4)
Metformin	3823 (54.8)
Sulfonylurea	2434 (34.9)
Insulin	4039 (57.9)
Antihypertensives, n (%)	6637 (95.1)
ACE inhibitors or ARBs	5655 (81.0)
β-blockers	4144 (59.4)
Diuretics	3711 (53.2)
Calcium antagonists	2870 (41.1)
Platelet aggregation inhibitors (excluding heparin), n (%)	4764 (68.3)
Statins, n (%)	5010 (71.8)

Data are mean ± SD unless otherwise specified, and may change slightly when the trial is completed

*ACE* angiotensin-converting enzyme, *ARB* angiotensin-receptor blocker, *BMI* body-mass index, *eGFR* estimated glomerular filtration rate, *HbA1c* glycated hemoglobin A1c, *HDL* high-density lipoprotein, *LDL* low-density lipoprotein, *MDRD* Modification of Diet in Renal Disease study equation, *UACR* urinary albumin-to-creatinine ratio

^a^American Indian/Alaska Native or Native Hawaiian/other Pacific Islander, ^b^ calculated by multiplying mg/dL values by 0.0555 [34], ^c^ n = 6977, ^d^ n = 6975, ^e^ n = 6915; ^f^ n = 6749, ^g^ n = 6744, ^h^ n = 6748

Mean levels of low-density lipoprotein cholesterol, high-density lipoprotein cholesterol and triglycerides were 2.4 mmol/L (91 mg/dL), 1.2 mmol/L (45 mg/dL), and 2.1 mmol/L (188 mg/dL), respectively. A total of 5010 (72%) and 4764 (68%) participants were taking statins and platelet aggregation inhibitors (excluding heparin), respectively. Almost all participants (n = 6802 [97%]) were taking glucose-lowering medication at baseline. Over half (n = 4039 [58%]) were taking insulin, while metformin was the most frequently used oral drug (n = 3823, [55%]) (Table 2).

**Fig. 4:**
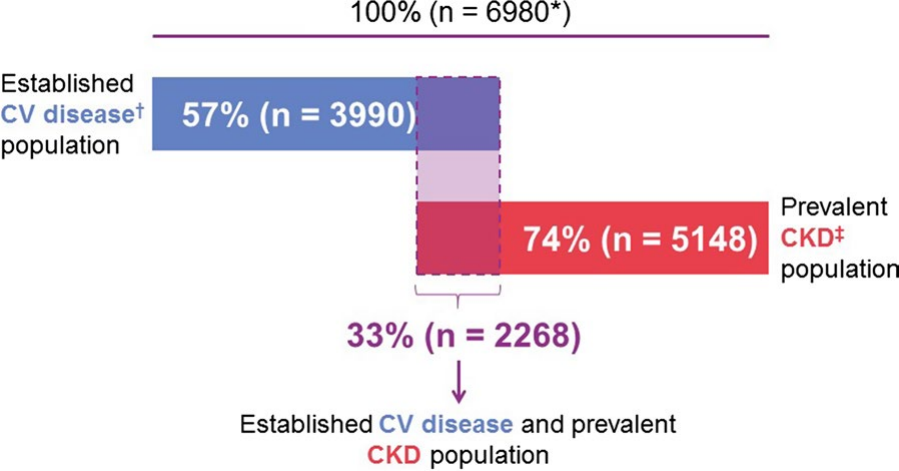
Proportion of patients included in the CARMELINA^®^ trial with established CV disease, prevalent kidney disease, or both. *110 patients without established CV disease had eGFR ≥ 60 mL/min/1.73 m^2^ and UACR ≤ 300 mg/g. ^†^Defined as albuminuria (UACR ≥ 30 mg/g or ≥ 30 μg albumin/min or ≥ 30 mg albumin/24 h) and prevalent macrovascular disease (≥ 1 of the following: confirmed history of myocardial infarction; advanced coronary artery disease; high-risk single-vessel coronary artery disease; history of ischemic or hemorrhagic stroke; presence of carotid artery disease; presence of peripheral artery disease). ^‡^Defined as eGFR < 60 mL/min/1.73 m^2^ or macroalbuminuria (UACR > 300 mg/g). *CKD* chronic kidney disease, *CV* cardiovascular, *eGFR* estimated glomerular filtration rate, *UACR* urinary albumin-to-creatinine ratio

The majority of patients from the overall trial population (5148 [74%]), had prevalent kidney disease at baseline, defined as eGFR < 60 mL/min/1.73 m^2^ or UACR ≥ 300 mg/g, while 3990 (57%) had established CV disease with elevated UACR (≥ 30 mg/g) and 2268 (32.5%) had both conditions (Fig. 4). Baseline characteristics for patients with prevalent kidney disease are shown in Table 3, while Additional files 2 and 3 show baseline characteristics for those with established CV disease and those with both prevalent kidney disease and established CV disease.

**Table 3:** Baseline characteristics by prevalent kidney disease at baseline

	Prevalent CKD^a^ (n = 5148)	No prevalent CKD (n = 1832)
Age, years	66.8 ± 9.0	62.9 ± 8.8
Male, n (%)	3114 (60.5)	1276 (69.7)
Race, n (%)		
White	4042 (78.5)	1553 (84.8)
Asian	508 (9.9)	133 (7.3)
Black/African American	349 (6.8)	62 (3.4)
Other^b^	249 (4.8)	84 (4.6)
Region, n (%)		
Europe	1997 (38.8)	836 (45.6)
South America	1658 (32.2)	652 (35.6)
North America	984 (19.1)	196 (10.7)
Asia	509 (9.9)	148 (8.1)
Smoking status, n (%)		
Never smoker	2821 (54.8)	932 (50.9)
Ex-smoker	1861 (36.1)	646 (35.3)
Current smoker	461 (9.0)	252 (13.8)
Missing	5 (0.1)	2 (0.1)
eGFR (MDRD), mL/min/1.73 m^2^	44.6 ± 19.2	82.6 ± 16.9
eGFR (MDRD), n (%)		
≥ 90 mL/min/1.73 m^2^	179 (3.5)	549 (30.0)
≥ 60 to < 90 mL/min/1.73 m^2^	620 (12.0)	1283 (70.0)
≥ 45 to < 60 mL/min/1.73 m^2^	1349 (26.2)	0
≥ 30 to < 45 mL/min/1.73 m^2^	1937 (37.6)	0
< 30 mL/min/1.73 m^2^	1063 (20.6)	0
UACR, mg/g, median (25th–75th percentile)	336 (60–1126)^d^	70 (27–138)^j^
UACR, n (%) [mg/g]		
< 30	907 (17.6)	483 (26.4)
30–300	1548 (30.1)	1348 (73.6)
> 300	2691 (52.3)	0
BMI, kg/m^2^	31.4 ± 5.4^e^	31.0 ± 5.1
HbA1c, %	7.9 ± 1.0	8.0 ± 1.0
Fasting plasma glucose, mmol/L^c^ (mg/dL)	8.3 ± 2.6 (150.1 ± 47.5)^f^	8.7 ± 5.0 (156.5 ± 90.3)^k^
Diabetes duration, years	15.9 ± 9.6	11.6 ± 8.4
Systolic blood pressure, mmHg	141.7 ± 18.5	137.2 ± 15.3
Diastolic blood pressure, mmHg	77.5 ± 10.8	78.8 ± 9.5
Total cholesterol, mmol/L (mg/dL)	4.5 ± 1.3 (173 ± 50)^g^	4.4 ± 1.2 (168 ± 44)^l^
LDL cholesterol, mmol/L (mg/dL)	2.4 ± 1.1 (92 ± 40)^h^	2.3 ± 1.0 (90 ± 37)^m^
HDL cholesterol, mmol/L (mg/dL)	1.2 ± 0.3 (45 ± 13)^i^	1.1 ± 0.3 (44 ± 12)^l^
Triglycerides, mmol/L (mg/dL)	2.2 ± 1.5 (191 ± 136)^g^	2.0 ± 1.4 (181 ± 125)^l^
Glucose-lowering therapy, n (%)	5013 (97.4)	1789 (97.7)
Metformin	2350 (45.6)	1473 (80.4)
Sulfonylurea	1653 (32.1)	781 (42.6)
Insulin	3294 (64.0)	745 (40.7)
Antihypertensives, n (%)	4946 (96.1)	1691 (92.3)
ACE inhibitors or ARBs	4183 (81.3)	1472 (80.3)
β-blockers	3043 (59.1)	1101 (60.1)
Diuretics	3032 (58.9)	679 (37.1)
Calcium antagonists	2313 (44.9)	557 (30.4)
Platelet aggregation inhibitors (excluding heparin), n (%)	3365 (65.4)	1399 (76.4)
Statins, n (%)	3708 (72.0)	1302 (71.1)

Data are mean ± SD unless otherwise specified, and may change slightly when the trial is completed

*ACE* angiotensin-converting enzyme, *ARB* angiotensin-receptor blocker, *BMI* body-mass index, *CKD* chronic kidney disease, *eGFR* estimated glomerular filtration rate, *HbA1c* glycated hemoglobin A1c, *HDL* high-density lipoprotein, *LDL* low-density lipoprotein, *MDRD* Modification of Diet in Renal Disease study equation, *UACR* urinary albumin-to-creatinine ratio

^a^Defined as eGFR < 60 mL/min/1.73 m^2^ or macroalbuminuria (UACR > 300 mg/g), ^b^ American Indian/Alaska Native or Native Hawaiian/other Pacific Islander, ^c^ calculated by multiplying mg/dL values by 0.0555 [34], ^d^ n = 5146, ^e^ n = 5143, ^f^ n = 5097, ^g^ n = 4971, ^h^ n = 4967, ^i^ n = 4970, ^j^ n = 1831, ^k^ n = 1818, ^l^ n = 1778, ^m^ n = 1777

**Fig. 5:**
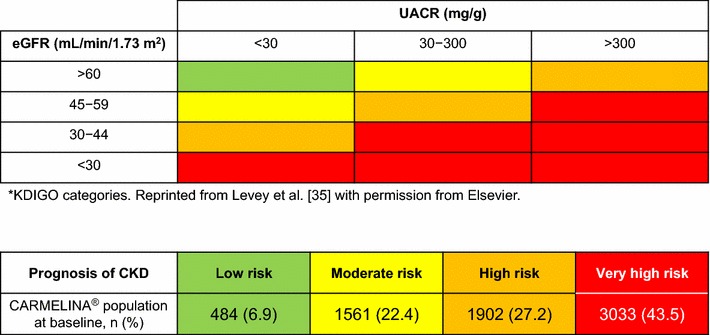
Prognosis of CKD in the CARMELINA^®^ trial population by eGFR and albuminuria categories.*CKD chronic kidney disease, *eGFR* estimated glomerular filtration rate, *KDIGO* Kidney Disease: Improving Global Outcomes, *UACR* urinary albumin-to-creatinine ratio

Approximately 71% of CARMELINA^®^ participants are considered at high risk (n = 1902 [27.2%]) or very high risk (n = 3033 [43.5%]) for adverse kidney events, on the basis of their eGFR and albuminuria status at baseline according to risk stratification by Kidney Disease: Improving Global Outcomes (KDIGO) foundation standards (Fig. 5).

## Discussion

The CARMELINA^®^ trial comprises 6991 patients with T2D randomized to daily oral treatment with the DPP-4 inhibitor linagliptin or placebo on top of standard care. Baseline characteristics of the treated set (n = 6980) delineate a population with long-standing T2D and a substantial burden of both CV and kidney disease. As intended, this will enable assessment of whether linagliptin is non-inferior—and, if so, superior—to placebo for CV and renal outcomes in individuals with T2D at very high vascular risk.

A pooled analysis of 19 randomized clinical trials of linagliptin involving 9459 individuals found no increased risk for major adverse CV events [27]. However, this analysis was limited by the short duration of drug exposure, inclusion of participants at relatively low CV risk, the small number of CV events for analysis, and the fact that these trials were not designed to assess either CV or renal safety. In hypothesis-generating, mechanistic clinical studies, linagliptin did not alter macrovascular function, but showed potential to improve microvascular function [36, 37], reduced early atherosclerotic vascular wall inflammation [38] and appeared to improve arterial stiffness [39].

In the last decade, a multitude of CV outcomes trials of glucose-lowering drugs have been initiated [29]. The trials completed to date have shown discordant results. The studies of empagliflozin, an SGLT2 inhibitor, and liraglutide, a GLP-1 receptor agonist, observed statistically significant reductions in CV mortality of 38% (hazard ratio, 0.62 [95% CI 0.49–0.77]) [9] and 22% (hazard ratio, 0.78 [95% CI 0.66–0.93]) [11], respectively. The outcomes trial of the SGLT2 inhibitor canagliflozin found a significant reduction in CV events (hazard ratio, 0.86 [95% CI 0.75–0.97) [13]. The GLP-1 receptor agonist semaglutide was shown to be non-inferior to placebo in CV safety (hazard ratio, 0.74, 95% CI 0.58–0.95) [12], while another two GLP-1 receptor agonists (lixisenatide [10] and once-weekly exenatide [15]) have demonstrated CV safety but no significant reduction in CV events. The three DPP-4 inhibitors studied to date (saxagliptin [6], alogliptin [7], sitagliptin [8]) have demonstrated neutral effects on atherosclerotic CV disease. However, a significantly increased risk of hospitalization for heart failure with saxagliptin was observed in the SAVOR-TIMI 53 study [6]. It remains to be determined whether this reflects a class effect of DPP-4 inhibitors, as a numerical (but non-significant) increase in hospitalization for heart failure was also seen in the EXAMINE study of alogliptin [23] but not in the TECOS study of sitagliptin [24]. Recent observational studies have suggested that individuals treated with DPP-4 inhibitors may have a lower risk for CV disease (including events of heart failure) than those treated with either a non-sulfonylurea insulin secretagogue or insulin [40], and that the risk of heart failure with linagliptin is not increased compared with sulfonylureas [41]. The CARMELINA^®^ study will thoroughly explore heart failure-related outcomes with linagliptin, as it includes hospitalization for heart failure as an adjudicated and pre-specified outcome and is proactively capturing information related to heart failure.

The CV outcomes data provided by CARMELINA^®^ will also complement the results from the ongoing CAROLINA^®^ study of linagliptin (ClinicalTrials.gov identifier: NCT01243424) [42]. CAROLINA^®^ is designed to evaluate the CV outcomes of linagliptin as a second-line treatment added to metformin. CAROLINA^®^ is comparing linagliptin with an active compound, the sulfonylurea glimepiride, rather than placebo, and with this unique design represents the only active-controlled, double-blind, multinational CV outcome study to date in patients with T2D. Furthermore, by design, the participants in CAROLINA^®^ have lower overall CV and renal risk, earlier stage of T2D disease (median duration 6.2 years), and better glycemic control (mean baseline HbA1c 7.2%) [42] than those in the CARMELINA^®^ study. The results from CAROLINA^®^ will help inform decision-making for second-line treatment of early T2D and may answer a long-standing question regarding the CV safety of sulfonylureas.

**Fig. 6:**
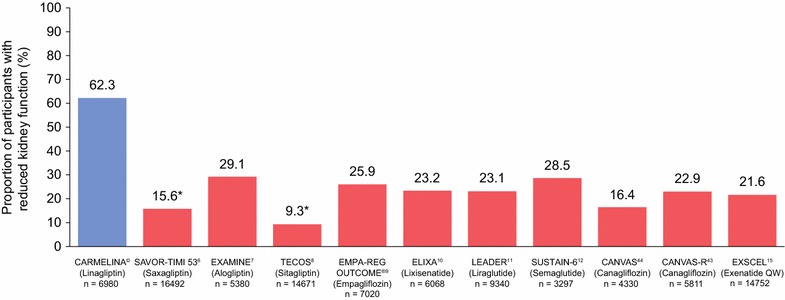
Proportion of patients with reduced kidney function at baseline (eGFR < 60 mL/min/1.73 m^2^) in CARMELINA^®^ compared to previously reported CV outcome trials of non-insulin glucose-lowering drugs for T2D. *eGFR < 50 mL/min/1.73 m^2^. *CV* cardiovascular, *eGFR* estimated glomerular filtration rate, *QW* once weekly, *T2D* type 2 diabetes

In addition to CV safety, CARMELINA^®^ was also designed and powered to evaluate renal outcomes of linagliptin treatment in an alpha-controlled manner—a notable feature compared to CV outcomes studies of other glucose-lowering drugs. Accordingly, the proportion of participants with overt kidney disease (defined as eGFR < 60 mL/min/1.73 m^2^ or macroalbuminuria [74%]) or reduced kidney function (eGFR < 60 mL/min/1.73 m^2^ [62%]) is substantially higher in CARMELINA^®^ than in other CV outcomes studies of oral glucose-lowering drugs in T2D (9.3–29.1%) [6–12, 43, 44] (Fig. 6). CARMELINA^®^ also includes a large number of individuals with very low eGFR (< 30 mL/min/1.73 m^2^) (n = 1063 [15%]) and/or elevated levels of albuminuria (n = 5587 [80%]) (Additional file 4).

No new pharmacotherapy for diabetic kidney disease has been licensed since the early 2000s when the angiotensin-receptor blockers losartan and irbesartan demonstrated efficacy in this indication [45]. Hypothesis-generating evidence has suggested that DPP-4 inhibitors may have specific renal effects independent of their glucose-lowering properties [46]. A pooled analysis of 13 randomized controlled studies found that linagliptin treatment was associated with a significant 16% reduction in the risk for clinically relevant adverse renal events (hazard ratio 0.84, 95% CI 0.72–0.97; *P* = 0.02) [28]. Additional data from pooled analyses of the linagliptin development program [47] and exploratory data from SAVOR-TIMI 53 and TECOS [48, 49] suggested that DPP-4 inhibitors could lower albuminuria in patients with T2D. This hypothesis was, however, not supported by the MARLINA-T2D™ study of linagliptin, which was the first randomized clinical study prospectively designed to investigate the effects of a DPP-4 inhibitor on albuminuria [50]. In MARLINA-T2D™, linagliptin elicited a placebo-adjusted, non-significant 6% reduction in albuminuria (95% CI − 15.0 to 3.0; *P* = 0.1954) after 24 weeks of treatment in individuals with T2D and early diabetic kidney disease who had residual albuminuria despite receiving ACE inhibitors or angiotensin-receptor blockers [50]. Since previous clinical evidence for renal effects of DPP-4 inhibitors has mainly emerged from patient populations at earlier stages of kidney disease, CARMELINA^®^ will answer an important question whether DPP-4 inhibition with linagliptin may have the potential to alter renal disease progression at more advanced stages of the renal continuum. Respective experimental evidence to support such a hypothesis has emerged from recent preclinical studies showing that linagliptin exerted anti-fibrotic, anti-inflammatory and anti-oxidant renal effects in animal models of diabetic kidney disease that—if translated to human disease—would be more likely to manifest as long-term disease-modifying renal effects than to elicit short-term changes in albuminuria [51–54].

## Conclusions

The CARMELINA^®^ trial is designed to assess CV and kidney outcomes of the DPP-4 inhibitor linagliptin versus placebo when added to standard care in individuals with T2D and established CV and/or kidney complications. Compared with the spectrum of CV outcome trials conducted in patients with T2D to date, CARMELINA^®^ has the highest number of individuals with prevalent kidney disease, including a large proportion of patients with severe kidney impairment and/or elevated albuminuria. These individuals are at a very high CV risk, face limited glucose-lowering treatment options, and have been largely underrepresented in previous CV outcomes trials in T2D. CARMELINA^®^ will thus enable assessment of the inherent effects of linagliptin on CV and kidney events in a vulnerable population at high cardio-renal risk. Results are expected in 2018.

## Additional files

**Additional file 1.** Full inclusion and exclusion criteria for CARMELINA^®^.

**Additional file 2.** Baseline characteristics by established CV disease at baseline in CARMELINA^®^ participants.

**Additional file 3.** Baseline characteristics by established CV disease and prevalent kidney disease at baseline in CARMELINA^®^ participants.

**Additional file 4.** CARMELINA^®^ baseline characteristics versus TECOS and SAVOR-TIMI 53.

## Abbreviations

3P-MACE: 3-point major adverse cardiovascular events
4P-MACE: 4-point major adverse cardiovascular events
ACE: angiotensin-converting enzyme
ARB: angiotensin-receptor blocker
BMI: body-mass index
CABG: coronary artery bypass graft surgery
CANVAS: CANagliflozin cardioVascular Assessment Study
CANVAS-R: CANVAS-Renal
CARMELINA^®^: CArdiovascular safety and Renal Microvascular outcomE study with LINAgliptin
CAROLINA^®^: CARdiovascular Outcome Study of LINAgliptin versus Glimepiride in Patients with Type 2 Diabetes
CI: confidence interval
CKD: chronic kidney disease
CT: computed tomography
CV: cardiovascular
CVD: cardiovascular disease
DPP-4: dipeptidyl peptidase-4
ECG: electrocardiogram
eGFR: estimated glomerular filtration rate
ELIXA: Evaluation of Lixisenatide in Acute Coronary Syndrome
EMA: European Medicines Agency
EMPA-REG OUTCOME: EMPAgliflozin Removal of Excess Glucose: Cardiovascular OUTCOME Event Trial in Type 2 Diabetes Mellitus Patients
ESKD: end-stage kidney disease
EXAMINE: Examination of Cardiovascular Outcomes with Alogliptin versus Standard of Care
EXSCEL: Exenatide Study of Cardiovascular Event Lowering
FDA: US Food and Drug Administration
GLP-1: glucagon-like peptide-1
HbA1c: glycated hemoglobin A1c
HDL: high-density lipoprotein
KDIGO: Kidney Disease: Improving Global Outcomes
LDL: low-density lipoprotein
LEADER: Liraglutide Effect and Action in Diabetes: Evaluation of Cardiovascular Outcome Results
MARLINA-T2D™: Efficacy, Safety & Modification of Albuminuria in Type 2 Diabetes Subjects With Renal Disease With LINAgliptin
MDRD: Modification of Diet in Renal Disease study equation
MI: myocardial infarction
MRI: magnetic resonance imaging
PCI: percutaneous coronary intervention
P-gp: P-glycoprotein
UACR: urinary albumin-to-creatinine ratio
SAVOR-TIMI 53: Saxagliptin Assessment of Vascular Outcomes Recorded in Patients with Diabetes Mellitus-Thrombolysis in Myocardial Infarction 53
SGLT2: sodium-glucose cotransporter 2
SUSTAIN-6: Trial to Evaluate Cardiovascular and Other Long-term Outcomes with Semaglutide in Subjects with Type 2 Diabetes
T2D: type 2 diabetes
TECOS: Trial Evaluating Cardiovascular Outcomes with Sitagliptin
UACR: urinary albumin-to-creatinine ratio
ULN: upper limit of normal
