# Supplementary material for: Rationale, design, and baseline characteristics of the CArdiovascular safety and Renal Microvascular outcomE study with LINAgliptin (CARMELINA®): a randomized, double-blind, placebo-controlled clinical trial in patients with type 2 diabetes and high cardio-renal risk
Source: Cardiovasc Diabetol. 2018 Mar 14;17:39. doi: 10.1186/s12933-018-0682-3 (PMC5870815; doi:10.1186/s12933-018-0682-3)
Supplement: Supplementary file 1 — Additional file 1. Full inclusion and exclusion criteria for CARMELINA®. [file 12933_2018_682_MOESM1_ESM.docx]

**Additional file 1. Full inclusion and exclusion criteria for CARMELINA^®^**

**Main diagnosis for study entry**

Patients with documented diagnosis of type 2 diabetes mellitus (T2DM) at high risk of CV events and meeting all other eligibility criteria can be enrolled in the study.

**Inclusion criteria**

1) Documented diagnosis of T2DM before visit 1 (screening).

2) Male or female patients who are drug-naïve or pre-treated with any antidiabetic background therapy, excluding treatment with GLP-1 receptor agonists, DPP-4 inhibitors or SGLT-2 inhibitors if ≥ 7 consecutive days.

3) Stable antidiabetic background medication (unchanged daily dose) for at least 8 weeks prior to randomization. If insulin is part of the background therapy, the average daily insulin dose should not have been changed by more than 10% within the 8 weeks prior to randomization compared with the daily insulin dose at randomization.

4) HbA1c of ≥ 6.5% and ≤ 10.0% at visit 1 (screening).

5) Age **≥** 18 years at visit 1 (screening). *For Japan only: Age* ***≥*** *20 years at Visit 1.*

6) Body Mass Index (BMI) ≤ 45 kg/m2 at visit 1 (screening).

7) Signed and dated written informed consent by date of visit 1 (screening) in accordance with GCP and local legislation prior to any study related procedure.

8) High risk of CV events (**I and/or II**):

| **I**. |  | **Albuminuria (UACR ≥ 30 mg/g creatinine or ≥ 30 µg/min [microgram albumin per minute] or ≥ 30 mg/24 h [milligram albumin per 24 hours] in two out of three unrelated spot urine or timed samples in the last 24 months prior to randomization)* AND previous macrovascular disease, defined as either one or more:** |
| --- | --- | --- |
|  | a | Confirmed history of MI (> 2 months prior to Visit 1) |
|  | b | Advanced coronary artery disease, defined by any one of the following:  • ≥ 50% narrowing of the luminal diameter in 2 or more major coronary arteries by coronary angiography, MRI angiography or CT angiography;  *Definition of major coronary arteries: LAD (Left Anterior Descending). CX (Circumflex) or RCA (right coronary artery)*  • Left main stem coronary artery with ≥ 50% narrowing of the luminal diameter by coronary angiography, MRI angiography or CT angiography;  • Prior percutaneous or surgical revascularization of ≥ 2 major coronary arteries at least 2 months prior to Visit 1 (screening);  • The combination of prior percutaneous or surgical revascularization of 1 major coronary artery at least 2 months prior to visit 1 (screening), and ≥ 50% narrowing of the luminal diameter by coronary angiography, MRI angiography or CT angiography of at least 1 additional major coronary artery. |
|  | c | High-risk single-vessel coronary artery disease, defined as the presence of ≥ 50%  narrowing of the luminal diameter of one major coronary artery by coronary angiography, MRI angiography or CT angiography in patients not revascularised**:**  **AND** at least one of the following:  • A positive non invasive stress test, confirmed by either:  o a positive ECG exercise tolerance test in patients without left bundle branch block, Wolff-Parkinson-White syndrome, left ventricular hypertrophy with repolarization abnormality, or paced ventricular rhythm, atrial fibrillation in case of abnormal ST-T segments;  o a positive stress echocardiogram showing induced regional systolic wall motion abnormalities;  o a positive nuclear myocardial perfusion imaging stress test showing stress- induced reversible perfusion abnormality;  o a positive cardiac stress perfusion MRI showing a stress induced perfusion defect;  • Patient discharged from hospital with a documented diagnosis of unstable angina pectoris between 2 and 12 months prior to visit 1 (screening). |
|  | d | History of ischemic or haemorrhagic stroke (>3 months prior to visit 1) |
|  | e | Presence of carotid artery disease (symptomatic or not) documented by either:  o imaging techniques with at least one lesion estimated to be ≥50%  narrowing of the luminal diameter;  o prior percutaneous or surgical carotid revascularization. |
|  | f | Presence of peripheral artery disease documented by either:  o previous limb angioplasty, stenting or bypass surgery;  o previous limb or foot amputation due to macrocirculatory insufficiency;  o angiographic evidence of peripheral artery stenosis ≥ 50% narrowing of the luminal diameter in at least one limb (definition of peripheral artery: common iliac artery, internal iliac artery, external iliac artery, femoral artery, popliteal artery). |
| **II.** |  | **Evidence of impaired renal function with predefined UACR, with or without CV co- morbidities, defined as follows (and/or criteria):**  • Impaired renal function (as defined by MDRD formula) with an eGFR:15- **<**45 mL/min/1.73 m2 at visit 1 (screening) with any UACR.  • Impaired renal function (as defined by MDRD formula) with an eGFR ≥ 45-75 mL/min/1.73 m2 at visit 1 (screening) with an UACR > 200 mg/g creatinine or  > 200 µg/min (microgram albumin per minute) or > 200 mg/24 h [milligram albumin per 24 hours] demonstrated in two out of three unrelated spot urine or timed samples in the last 24 months prior to randomization. |

**Exclusion criteria**

1) Type 1 diabetes mellitus.

2) Treatment (≥ 7 consecutive days) with GLP-1 receptor agonists, other DPP-4 inhibitors or

SGLT-2 inhibitors prior to informed consent. Note: This also includes clinical trials where these antidiabetic drugs have been provided to the patient.

3) Active liver disease or impaired hepatic function, defined by serum levels of either ALT (SGPT), AST (SGOT), or alkaline phosphatase (AP) ≥3 x upper limit of normal (ULN) as determined at Visit 1.

4) eGFR <15 ml/min/1.73 m2 (severe renal impairment or ESRD, MDRD formula), as determined during screening at Visit 1 and/or the need for maintenance dialysis.

5) Any previous (or planned within next 12 months) bariatric surgery (open or laparoscopic)

or intervention (gastric sleeve).

6) Pre-planned coronary artery re-vascularisation (PCI, CABG) or any previous PCI and/or

CABG ≤ 2 months prior informed consent

7) Known hypersensitivity or allergy to the investigational products or its excipients.

8) Any previous or current alcohol or drug abuse that would interfere with trial participation in the opinion of the investigator.

9) Participation in another trial with an investigational drug ongoing or within 2 months prior to visit 1 (screening)*.

10) Pre-menopausal women (last menstruation ≤ 1 year prior to informed consent) who:

- are nursing or pregnant,

- or are of child-bearing potential and are not practicing an acceptable method of birth control (acceptable methods of birth control include tubal ligation, transdermal patch, intra uterine devices/systems (IUDs/IUSs), oral, implantable or injectable contraceptives, sexual abstinence (if allowed by local authorities), double barrier method and vasectomised partner) or do not plan to continue using acceptable method of birth control throughout the study and do not agree to submit to periodic pregnancy testing during participation in the trial.

11) Patients considered unreliable by the investigator concerning the requirements for follow- up during the study and/or compliance with study drug administration, have a life expectancy less than 5 years for non-CV causes, or have cancer other than non-melanoma skin cancer within last 3 years, or has any other condition than mentioned which in the opinion of the investigator, would not allow safe participation in the study.

12) Acute coronary syndrome (ACS), diagnosed ≤ 2 months prior to visit 1 (screening).

13) Stroke or TIA ≤ 3 months prior to visit 1 (screening).
