# Supplementary material for: Rationale, design, and baseline characteristics of the CArdiovascular safety and Renal Microvascular outcomE study with LINAgliptin (CARMELINA®): a randomized, double-blind, placebo-controlled clinical trial in patients with type 2 diabetes and high cardio-renal risk
Source: Cardiovasc Diabetol. 2018 Mar 14;17:39. doi: 10.1186/s12933-018-0682-3 (PMC5870815; doi:10.1186/s12933-018-0682-3)
Supplement: Supplementary file 2 — Additional file 2. Baseline characteristics by established CV disease at baseline in CARMELINA® participants. [file 12933_2018_682_MOESM2_ESM.docx]

**Additional file 2. Baseline characteristics by established CV disease at baseline in CARMELINA^®^ participants**

|  | **CVD***  **(n = 3990)** | **No CVD**  **(n = 2986)** |
| --- | --- | --- |
| Age, years | 64.8 ± 8.8 | 67.1 ± 9.2 |
| Male, n (%) | 2752 (69.0) | 1635 (54.8) |
| Race, n (%) |  |  |
| White | 3313 (83.0) | 2278 (76.3) |
| Asian | 334 (8.4) | 307 (10.3) |
| Black/African American | 163 (4.1) | 248 (8.3) |
| Other^†^ | 180 (4.5) | 153 (5.1) |
| Region, n (%) |  |  |
| Europe | 1713 (42.9) | 1119 (37.5) |
| South America | 1362 (34.1) | 947 (31.7) |
| North America | 546 (13.7) | 632 (21.2) |
| Asia | 369 (9.2) | 288 (9.6) |
| Smoking status, n (%) |  |  |
| Never smoker | 1994 (50.0) | 1756 (58.8) |
| Ex-smoker | 1513 (37.9) | 993 (33.3) |
| Current smoker | 478 (12.0) | 235 (7.9) |
| Missing | 5 (0.1) | 2 (0.1) |
| eGFR (MDRD), mL/min/1.73 m^2^ | 66.3 ± 24.8 | 38.9 ± 14.5 |
| eGFR (MDRD), n (%) |  |  |
| ≥90 mL/min/1.73 m^2^ | 714 (17.9) | 14 (0.5) |
| ≥60−<90 mL/min/1.73 m^2^ | 1639 (41.1) | 263 (8.8) |
| ≥45−<60 mL/min/1.73 m^2^ | 790 (19.8) | 558 (18.7) |
| ≥30−<45 mL/min/1.73 m^2^ | 598 (15.0) | 1338 (44.8) |
| <30 mL/min/1.73 m^2^ | 249 (6.2) | 813 (27.2) |
| UACR, mg/g, median (25^th^−75^th^ percentile) | 135 (50−497)^§^ | 243 (33−1138)^††^ |
| UACR, n (%) |  |  |
| <30 mg/g | 668 (16.7) | 722 (24.2) |
| 30−300 mg/g | 2010 (50.4) | 885 (29.6) |
| >300 mg/g | 1311 (32.9) | 1377 (46.1) |
| BMI, kg/m^2^ | 31.0 ± 5.1^§^ | 31.7 ± 5.6^‡‡^ |
| HbA1c, % | 8.0 ± 1.0 | 7.9 ± 1.0 |
| Fasting plasma glucose, mmol/L^‡^ (mg/dL) | 8.5 ± 3.7 (153.7 ± 66.7)^¶^ | 8.3 ± 3.0 (149.2 ± 54.3)^§§^ |
| Diabetes duration, years | 13.8 ± 9.3 | 15.9 ± 9.6 |
| Systolic blood pressure, mmHg | 139.2 ± 16.8 | 142.2 ± 19.1 |
| Diastolic blood pressure, mmHg | 78.2 ± 10.2 | 77.4 ± 10.8 |
| Total cholesterol, mmol/L (mg/dL) | 4.4 ± 1.2 (169 ± 48)^ǁ^ | 4.6 ± 1.3 (176 ± 49)^¶¶^ |
| LDL cholesterol, mmol/L (mg/dL) | 2.3 ± 1.0 (89 ± 39)** | 2.4 ± 1.0 (94 ± 40)^ǁǁ^ |
| HDL cholesterol, mmol/L (mg/dL) | 1.1 ± 0.3 (44 ± 12)^ǁ^ | 1.2 ± 0.4 (45 ± 14)*** |
| Triglycerides, mmol/L (mg/dL) | 2.1 ± 1.5 (185 ± 134)^ǁ^ | 2.2 ± 1.5 (193 ± 132)^¶¶^ |
| Glucose-lowering therapy, n (%) | 3904 (97.8) | 2894 (96.9) |
| Metformin | 2657 (66.6) | 1162 (38.9) |
| Sulfonylurea | 1486 (37.2) | 945 (31.6) |
| Insulin | 2077 (52.1) | 1960 (65.5) |
| Antihypertensives, n (%) | 3764 (94.3) | 2869 (96.1) |
| ACE inhibitors or ARBs | 3214 (80.6) | 2437 (81.6) |
| β-blockers | 2563 (64.2) | 1578 (52.8) |
| Diuretics | 1848 (46.3) | 1861 (62.3) |
| Calcium antagonists | 1453 (36.4) | 1413 (47.3) |
| Platelet aggregation inhibitors (excluding heparin), n (%) | 3141 (78.7) | 1621 (54.3) |
| Statins, n (%) | 2979 (74.7) | 2028 (67.9) |
| Data are mean ± SD unless otherwise specified, and may change slightly when the trial is completed. Participants with missing CVD status: n = 4  *ACE* angiotensin-converting enzyme, *ARB* angiotensin-receptor blocker, *BMI* body-mass index, *CVD* cardiovascular disease, *eGFR* estimated glomerular filtration rate, *HbA1c* glycated hemoglobin A1c, *HDL* high-density lipoprotein, *LDL* low-density lipoprotein, *MDRD* Modification of Diet in Renal Disease study equation, *UACR* urinary albumin-to-creatinine ratio  *Defined according to study inclusion criteria as albuminuria (UACR ≥30 mg/g or ≥30 μg albumin/min or ≥30 mg albumin/24 h) and prevalent macrovascular disease (one or more of the following: confirmed history of myocardial infarction; advanced coronary artery disease; high-risk single-vessel coronary artery disease; history of ischemic or hemorrhagic stroke; presence of carotid artery disease; presence of peripheral artery disease), ^†^American Indian/Alaska Native or Native Hawaiian/other Pacific Islander, ^‡^calculated by multiplying mg/dL values by 0.0555 [[52](#_ENREF_52)], ^§^n = 3989, ^¶^n = 3953, ^ǁ^n = 3867, **n = 3865, ^††^n = 2984, ^‡‡^n = 2982, ^§§^n = 2958, ^¶¶^n = 2878, ^ǁǁ^n = 2875, ***n = 2877 | | |
