# Supplementary material for: Rationale, design, and baseline characteristics of the CArdiovascular safety and Renal Microvascular outcomE study with LINAgliptin (CARMELINA®): a randomized, double-blind, placebo-controlled clinical trial in patients with type 2 diabetes and high cardio-renal risk
Source: Cardiovasc Diabetol. 2018 Mar 14;17:39. doi: 10.1186/s12933-018-0682-3 (PMC5870815; doi:10.1186/s12933-018-0682-3)
Supplement: Supplementary file 3 — Additional file 3. Baseline characteristics by established CV disease and prevalent kidney disease at baseline in CARMELINA® participants. [file 12933_2018_682_MOESM3_ESM.docx]

**Additional file 3. Baseline characteristics by established CV disease and prevalent kidney disease at baseline in CARMELINA^®^ participants**

|  | **CVD* + CKD^†^**  **(n = 2268)** | **CVD^*^ only**  **(n = 1722)** |
| --- | --- | --- |
| Age, years | 66.2 ± 8.6 | 62.9 ± 8.8 |
| Male, n (%) | 1538 (67.8) | 1214 (70.5) |
| Race, n (%) |  |  |
| White | 1852 (81.7) | 1461 (84.8) |
| Asian | 205 (9.0) | 129 (7.5) |
| Black/African American | 108 (4.8) | 55 (3.2) |
| Other^‡^ | 103 (4.5) | 77 (4.5) |
| Region, n (%) |  |  |
| Europe | 928 (40.9) | 785 (45.6) |
| South America | 737 (32.5) | 625 (36.3) |
| North America | 377 (16.6) | 169 (9.8) |
| Asia | 226 (10.0) | 143 (8.3) |
| Smoking status, n (%) |  |  |
| Never smoker | 1135 (50.0) | 859 (49.9) |
| Ex-smoker | 896 (39.5) | 617 (35.8) |
| Current smoker | 234 (10.3) | 244 (14.2) |
| Missing | 3 (0.1) | 2 (0.1) |
| eGFR (MDRD), mL/min/1.73 m^2^ | 53.5 ± 22.0 | 83.2 ± 17.0 |
| eGFR (MDRD), n (%) |  |  |
| ≥90 mL/min/1.73 m^2^ | 177 (7.8) | 537 (31.2) |
| ≥60−<90 mL/min/1.73 m^2^ | 454 (20.0) | 1185 (68.8) |
| ≥45−<60 mL/min/1.73 m^2^ | 790 (34.8) | 0 |
| ≥30−<45 mL/min/1.73 m^2^ | 598 (26.4) | 0 |
| <30 mL/min/1.73 m^2^ | 249 (11.0) | 0 |
| UACR, mg/g, median (25^th^−75^th^ percentile) | 399 (93−1059) | 70 (28−133)^‡‡^ |
| UACR, n (%) |  |  |
| <30 mg/g | 224 (9.9) | 444 (25.8) |
| 30−300 mg/g | 733 (32.3) | 1277 (74.2) |
| >300 mg/g | 1311 (57.8) | 0 |
| BMI, kg/m^2^ | 31.0 ± 5.2^¶^ | 30.9 ± 5.0 |
| HbA1c, % | 8.0 ± 1.0 | 8.0 ± 1.0 |
| Fasting plasma glucose, mmol/L^§^ (mg/dL) | 8.4 ± 2.5 (152.0 ± 45.7)^ǁ^ | 8.7 ± 4.8 (156.0 ± 86.9)^§§^ |
| Diabetes duration, years | 15.6 ± 9.5 | 11.6 ± 8.4 |
| Systolic blood pressure, mmHg | 140.8 ± 17.7 | 137.2 ± 15.2 |
| Diastolic blood pressure, mmHg | 77.7 ± 10.6 | 78.8 ± 9.5 |
| Total cholesterol, mmol/L (mg/dL) | 4.4 ± 1.3 (170 ± 50)** | 4.3 ± 1.2 (167 ± 44)^¶¶^ |
| LDL cholesterol, mmol/L (mg/dL) | 2.3 ± 1.1 (90 ± 41)^††^ | 2.3 ± 1.0 (89 ± 37)^ǁǁ^ |
| HDL cholesterol, mmol/L (mg/dL) | 1.1 ± 0.3 (44 ± 12)** | 1.1 ± 0.3 (44 ± 12)^¶¶^ |
| Triglycerides, mmol/L (mg/dL) | 2.1 ± 1.6 (189 ± 140)** | 2.0 ± 1.4 (180 ± 125)^¶¶^ |
| Glucose-lowering therapy, n (%) | 2221 (97.9) | 1683 (97.7) |
| Metformin | 1270 (56.0) | 1387 (80.5) |
| Sulfonylurea | 746 (32.9) | 740 (43.0) |
| Insulin | 1375 (60.6) | 702 (40.8) |
| Antihypertensives, n (%) | 2172 (95.8) | 1592 (92.5) |
| ACE inhibitors or ARBs | 1828 (80.6) | 1386 (80.5) |
| β-blockers | 1512 (66.7) | 1051 (61.0) |
| Diuretics | 1217 (53.7) | 631 (36.6) |
| Calcium antagonists | 933 (41.1) | 520 (30.2) |
| Platelet aggregation inhibitors (excluding heparin), n (%) | 1801 (79.4) | 1340 (77.8) |
| Statins, n (%) | 1744 (76.9) | 1235 (71.7) |
| Data are mean ± SD unless otherwise specified, and may change slightly when the trial is completed  *ACE* angiotensin-converting enzyme, *ARB* angiotensin-receptor blocker, *BMI* body-mass index, *CVD* cardiovascular disease, *eGFR* estimated glomerular filtration rate, *HbA1c* glycated hemoglobin A1c, *HDL* high-density lipoprotein, *LDL* low-density lipoprotein, *MDRD* Modification of Diet in Renal Disease study equation, *UACR* urinary albumin-to-creatinine ratio  *Defined according to study inclusion criteria as albuminuria (UACR ≥30 mg/g or ≥30 μg albumin/min or ≥30 mg albumin/24 h) and prevalent macrovascular disease (one or more of the following: confirmed history of myocardial infarction; advanced coronary artery disease; high-risk single-vessel coronary artery disease; history of ischemic or hemorrhagic stroke; presence of carotid artery disease; presence of peripheral artery disease), ^†^Defined as eGFR <60 mL/min/1.73 m^2^ or macroalbuminuria (UACR >300 mg/g), ^‡^American Indian/Alaska Native or Native Hawaiian/other Pacific Islander, ^§^calculated by multiplying mg/dL values by 0.0555 [[52](#_ENREF_52)], ^¶^n = 2267, ^ǁ^n = 2245, **n = 2198, ^††^n = 2197, ^‡‡^n = 1721, ^§§^n = 1708, ^¶¶^n = 1669, ^ǁǁ^n = 1668 | | |
